# Liver hydatid cyst perforated into the large bowel: a case report

**DOI:** 10.1186/1757-1626-2-6999

**Published:** 2009-05-18

**Authors:** Ioannis G Bougioukas, Nikolaos Courcoutsakis, Odysseas S Korakianitis, Antonios-Apostolos K Tentes, Panagiotis Prasopoulos

**Affiliations:** 1 Surgical Department, Didimotichon General HospitalKonstantinoupoleos 1 Str. 68300, DidimotichonGreece; 2 Department of Radiology, Democritus University, Alexandroupolis General HospitalDragana 68100, AlexanroupolisGreece; 3 Department of Anesthesiology, Didimotichon General HospitalKonstantinoupoleos 1 Str. 68300, DidimotichonGreece

## Abstract

Perforation of the liver hydatid cyst into hollow abdominal organs is an extremely rare complication. A case of two synchronous hydatid cysts in an old lady is presented. The patient had one multilobular cyst perforated into the right colon and another one uncomplicated located at the right ovary. She underwent partial cystectomy, omentoplasty, right hemicolectomy, and total hysterectomy, and had an uneventful recovery.

## Introduction

Echinococcosis is a parasitosis endemic in Mediterranean countries, the Middle and Far East, South America, Australia, and East Africa. The most frequent complications of liver hydatid cysts include those related to the compression of adjacent organs or to perforation into the biliary tree, pleural, or pericardial cavity, or even to cyst infection [1]. Direct perforation of the cyst into hollow abdominal organs is very unusual. Communication of the cyst with the duodenum [2,3], the stomach [4], and the left colon [5] has been reported. Perforation into the right colon has not been previously reported to the best of our knowledge.

## Case presentation

**Figure 1.**
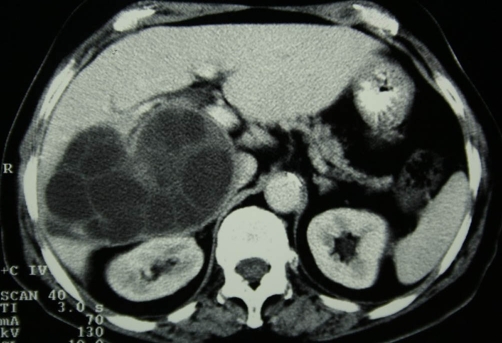
Contrast enhanced CT section at the level of the right subhepatic space. A large multilobular cystic mass of the right liver lobe is present extending to the subhepatic space.

**Figure 2.**
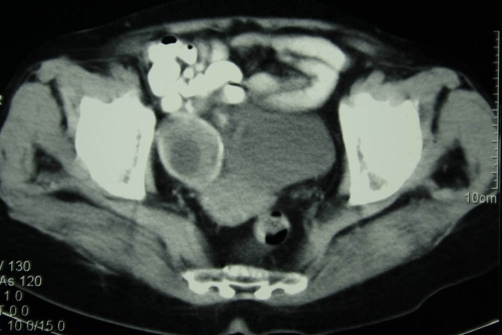
CT section at the level of the pelvis. A second cystic mass completely calcified is present at the right ovary.

A 76-year-old Greek woman was admitted to the hospital complaining of right upper quadrant abdominal pain. The ultrasound examination revealed gall-bladder lithiasis and a large multi-cystic mass compatible with hydatid cyst. The CT-scan confirmed the diagnosis of a large hydatid cyst of the right lobe of the liver extending to the subhepatic space (Figure 1) while a second cystic mass at the right ovary was identified (Figure 2). Indirect hemoagglutination echinococcosis test was positive but the patient denied surgery.

**Figure 3.**
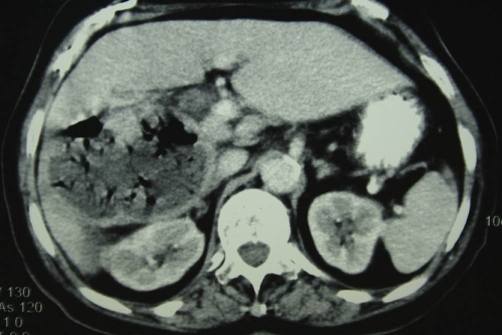
Contrast enhanced CT sections at approximately the level of the right subhepatic space. The right liver lobe cystic lesion contains multiple air-bubbles producing artifacts.

**Figure 4.**
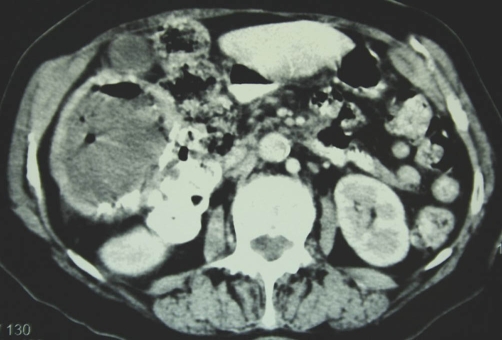
The subhepatic extension of the cyst is in contact with the right colon. The cystic wall exhibits coarse calcifications. The gall-bladder is entirely displaced by the cyst.

Two years later she was re-admitted because of high fever and acute pain at the right upper abdominal quadrant. The CT-scan demonstrated the hydatid cyst in segments V, VI, and VII of the liver containing multiple air-bubbles. The subhepatic extension of the cyst was in close relationship to the right colon (Figure 3). The second uncomplicated cyst at the right ovary was re-imaged without any significant change (Figure 4). In addition, uterine prolapse was found by physical examination.

**Figure 5.**
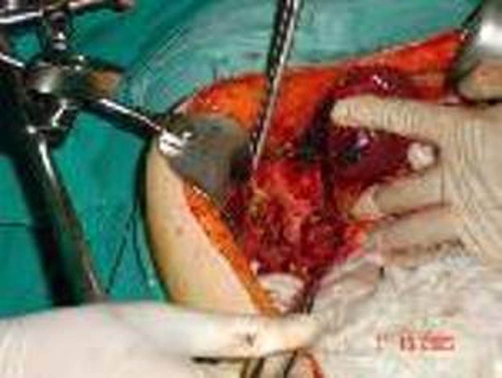
Intraoperative picture of the hydatid cyst that has already been drained and partially resected.

The patient underwent partial cystectomy, cholecystectomy, right hemicolectomy, omentoplasty (Figure 5), and total hysterectomy. The gastrointestinal tract was reconstructed with side-to-side ileocolic anastomosis. The patient had an uneventful recovery. Histopathology confirmed the clinical diagnosis.

## Discussion

The perforation of the cyst into the right colon is either secondary to infection of the cyst as probably occurred in this case or to primary pathology of the perforated organ. The content of the cyst did not drain into the large bowel because the fistula was very narrow and allowed only gas from the large bowel to penetrate into the cyst. Hydatid cyst of the ovaries is also very rare and a few cases have been reported in the literature [6,7]. Right hemicolectomy was considered less risky than simple suturing of the large bowel. Total hysterectomy was considered the operation of choice, once the patient had uterine prolapse and a cyst at the right ovary.

## List of abbreviation

CT: Computerized tomography
